# The Role of Musculoskeletal Ultrasound in Psoriatic Arthritis: From Preclinical Detection to Treatment Monitoring

**DOI:** 10.1007/s11926-026-01225-z

**Published:** 2026-06-12

**Authors:** Emilio D’Ignazio, Didem Sahin, Caterina Baldi, Paul Emery, Dennis McGonagle, Richard Wakefield, Helena Marzo-Ortega, Alen Zabotti, Emilio Filippucci, Katya Meridor, Andrea Di Matteo

**Affiliations:** 1 Department of Medicine, Surgery and Neurosciences, Rheumatology Unit, University of Siena, Siena, Italy; 2 Leeds Institute of Rheumatic and Musculoskeletal Medicine, University of Leeds, Leeds, UK; 3 NIHR Leeds Biomedical Research Centre, The Leeds Teaching Hospitals NHS Trust, Leeds, UK; 4 Rheumatology Clinic, Department of Medicine, University of Udine, c/o Azienda Sanitaria Universitaria Friuli Centrale, Udine, Italy; 5 Rheumatology Unit, Department of Clinical and Molecular Sciences, Polytechnic University of Marche, Jesi, Italy; 6 Rheumatology Department, Faculty of Medicine, Tel Aviv Sourasky Medical Centre, Tel Aviv, Israel

**Keywords:** Ultrasound, Psoriasis, Psoriatic arthritis, Pre-clinical disease, Early diagnosis, Monitoring, Joint, Tendon, Enthesis, Difficult to treat, Scoring system, Dactylitis.

## Abstract

**Purpose of Review:**

Psoriatic arthritis (PsA) is a chronic, immune-mediated inflammatory disease with highly heterogeneous clinical manifestations associated with psoriasis (PsO). The wide variability in presentation, together with the absence of definitive serological biomarkers, makes early diagnosis particularly challenging. This review evaluates the role of ultrasound in identifying and characterising musculoskeletal involvement across the psoriatic disease (PsD) continuum—from asymptomatic PsO to established PsA.

**Recent Findings:**

Ultrasound can detect subclinical synovitis, enthesitis, peritendonitis, tenosynovitis, bursitis, and structural damage in PsD. Evidence highlights its value in the early identification of musculoskeletal changes in patients with PsO who are at risk of progressing to PsA, with important implications for disease interception and prevention. Additional applications include differential diagnosis; assessment of enthesitis and distinction between inflammatory and non-inflammatory disease; and monitoring of therapeutic response, including in refractory disease. Ultrasound also demonstrates prognostic utility by detecting subclinical inflammation, predicting flares and future structural damage, and supporting personalized treatment strategies. Standardized ultrasound scoring systems and emerging methods for evaluating small hand entheses and dactylitis are also discussed.

**Summary:**

Ultrasound is an important tool for early detection, prognostic assessment, and management guidance in PsA, offering potential to prevent disease progression and inform precision medicine approach.

## Introduction

Psoriatic arthritis (PsA) is a chronic, immune-mediated inflammatory disease associated with, and often preceded by, skin psoriasis (PsO) [1]. It is clinically heterogeneous, affecting peripheral joints, the axial skeleton, and extra-articular sites such as entheses and peri-articular soft tissues [2, 3]. PsA can range from localised, oligoarticular, polyarticular to severe, refractory forms [4]. Without timely diagnosis and effective treatment, progressive joint damage, irreversible deformities, and functional impairment may occur [5].

In contrast to rheumatoid arthritis (RA), where diagnosis is aided by well-established serological markers such as anti-cyclic citrullinated peptide (anti-CCP) antibodies and rheumatoid factor (RF), PsA currently lacks definitive diagnostic biomarkers [6]. Further, conventional inflammatory markers, including C-reactive protein (CRP) and erythrocyte sedimentation rate (ESR), are not elevated in approximately 50% of people with PsA, further complicating disease detection and monitoring [1, 7]. While clinical and physical examinations remain central to assessing disease activity, they have limitations in detecting subtle inflammation and structural damage which can provide important information on disease progression and guide management [8, 9]. These diagnostic limitations highlight the importance of precise and reliable tools to assess psoriatic disease (PsD) across its full clinical spectrum, from the earliest, even preclinical phases in PsO to established PsA.

In recent years, ultrasound has gained significant prominence in the field of rheumatology due to its ability to provide non-invasive, real-time imaging of joints and surrounding soft tissue structures [10]. In PsA, ultrasound can detect subtle inflammatory changes such as synovitis, enthesitis, peri-tendonitis, tenosynovitis, and bursitis—even in the absence of overt clinical symptoms—as well as structural damage in joints (e.g., bone erosions, cartilage damage) and peri-articular soft tissues (e.g., tendinopathy, enthesopathy) not seen by radiography [11, 12].

Admittedly ultrasound cannot assess axial involvement that occurs in a substantial proportion of patients but such involvement typically accompanies peripheral joint disease and the vast majority of PsA cases present with peripheral manifestations [13]. Furthermore, diffuse peri-entheseal MRI determined osteitis is a lesion that is not directly visible on ultrasound and such lesions may contribute to bone pain in PsA . Finally, some deeper entheseal sites are inaccessible to ultrasound assessment, representing an additional limitation of the modality. 

 This review explores the evolving role of ultrasound across the ‘PsD continuum’. We discuss its use in detecting subclinical inflammatory changes during the preclinical phase in at-risk PsO patients and its contribution to early diagnosis by helping distinguish PsA from other types of arthritis. Furthermore, we examine the importance of ultrasound in ongoing disease monitoring and its critical role in managing treatment-refractory PsA cases, including differentiating patients with persistent inflammatory arthritis from those without imaging-detectable inflammation. We also highlight recent advances by organizations such as Outcome Measures in Rheumatology (OMERACT) and the Group for Research and Assessment of Psoriasis and Psoriatic Arthritis (GRAPPA) in developing standardized scoring methods, which promise to enhance the consistency and clinical utility of ultrasound assessments, ultimately improving patient outcomes.

## The Role of Ultrasound in Patients with PsO at Risk of PsA

PsO is a chronic skin disease affecting approximately 3% of the global population, and PsA develops in roughly 20–30% of patients with PsO over time [14]. Numerous studies have investigated the transition from PsO to PsA, leading to the introduction of the concept of the ‘PsD continuum’—a progressive model of disease development that mirrors the paradigm established for RA [15, 16].

In this framework, PsA is not typically viewed as the sudden onset of inflammatory arthritis but rather as a dynamic continuum that begins with a preclinical phase characterized by immune dysregulation and subclinical inflammation. This may progress through a symptomatic subclinical stage, where symptoms, such as arthralgia, fatigue, or stiffness, emerge before evolving into clinically apparent PsA.

International rheumatology organizations, including the European Alliance of Associations for Rheumatology (EULAR), have outlined three distinct phases in the transition from PsO to PsA [17, 18]:

1. *The increased risk phase*: This phase involves individuals with PsO who have additional risk factors, such as genetic predispositions (e.g., familiarity and specific HLA alleles) as well as obesity, nail PsO, and extensive skin involvement. These factors contribute to a long-term risk of developing PsA, which typically manifests 7–12 years after the onset of PsO.
2. *The subclinical phase*: Characterized by imaging-detected musculoskeletal inflammation and/or arthralgia, this phase represents a short-term risk of PsA, generally up to 1–3 years.
3. *The clinical phase*: Involves individuals with PsO and clinically evident synovitis. They should be considered to have PsA, when alternative diagnoses are excluded.

While progression generally increases through these stages, it is not always linear. For instance, some individuals with PsO and arthralgia may experience symptom regression, while others may develop symptoms even as imaging abnormalities resolve.

A deep understanding of this continuum is essential not only for unravelling the pathogenesis of PsA but also for identifying individuals at high risk [19]. Early identification of these subjects is paramount, as it opens the door to preventive strategies, ranging from lifestyle modifications to pharmacological interventions, that could potentially delay or even prevent the onset of PsA in at-risk populations [20, 21].

**Fig. 1:**
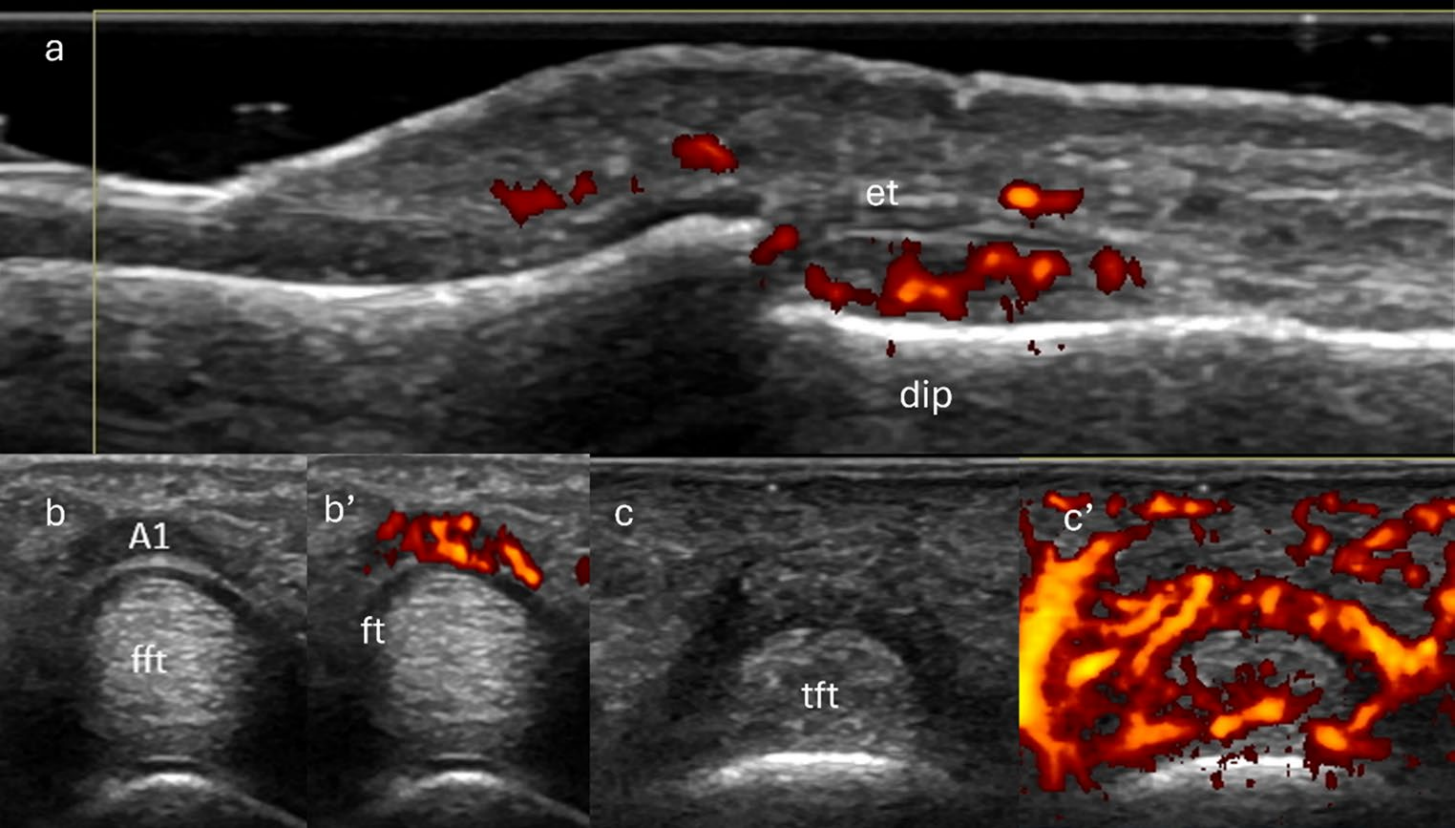
Ultrasound detection of subclinical inflammation in patients with psoriasis at risk of psoriatic arthritis. Legend. (**a**) Synovitis in the distal interphalangeal joint (DIP) joint of the finger with synovial hypertrophy and power Doppler signal (dorsal longitudinal scan); the image also shows enthesitis of the tendon insertion into the distal phalanx with entheseal power Doppler signal. (**b**) Grey-scale transverse volar scan of the A1 pulley demonstrates severe thickening with hypoechoic appearance, accompanied by Power Doppler (**b’**) signal. (**c**) Grey-scale image of tenosynovitis in the flexor tendons of the 2nd toe with synovial hypertrophy and active inflammation (the latter demonstrated by the presence of power Doppler signal) **(c’)** (volar transverse scan). Acronyms: dip: distal interphalangeal; et: extensor tendon; fft: finger flexor tendons; tft: toe flexor tendons

Within the pre-clinical context, ultrasound plays a crucial role in detecting subtle signs of musculoskeletal inflammation in areas that are difficult to assess clinically, including the distal interphalangeal (DIP) joints of the hands and toes, pulleys, and small tendons (Fig. 1).

Several studies have highlighted the utility of ultrasound for detecting subclinical inflammation in PsO patients [22]. A seminal study by Gisondi and colleagues demonstrated subclinical enthesopathy in patients with PsO, reporting significantly higher Glasgow Ultrasound Enthesitis Scoring System (GUESS) scores— an ultrasound based validated tool for assessing enthesitis—in asymptomatic PsO patients compared with healthy controls (HCs) (*p* < 0.001) [23]. Subsequently, another study found that 85% of PsO patients showed signs of synovitis versus 55% of HCs, with active synovitis observed exclusively in the PsO group (27.5% vs. 0%, *p* = 0.01) [24]. A further study comparing PsO with arthralgia (PsOAr), asymptomatic PsO, and HCs reported higher rates of ultrasound-detected tenosynovitis in PsOAr patients (29.5%) than in asymptomatic PsO (5.3%) or HCs (3.5%) (*p* < 0.01) [25].

Longitudinal studies suggest that ultrasound-detected inflammation may predict PsA development. In a two-year prospective study of 109 PsO patients and 90 HCs, ultrasound-detected synovitis and enthesitis were significant predictors of PsA (*p* < 0.001) [26]. Similarly, higher baseline GUESS scores were associated with concomitant synovitis and an increased risk of progression to PsA [27]. In a study of 102 patients (54 with PsOAr and 48 with PsO), those with active enthesitis detected by ultrasound at baseline were significantly more likely to develop PsA over an average follow-up of 309 days (*p* = 0.03) [25]. Baseline synovitis or tenosynovitis scores, however, did not correlate significantly with PsA progression. Alongside imaging findings, clinical features also showed strong predictive value, particularly when combined with ultrasound data. In a longitudinal study of 384 PsO patients followed for a mean of 33.0 ± 20.9 months, subclinical PsA, as defined by arthralgia, conferred a markedly higher risk of progression compared with PsO alone (hazard ratio [HR] = 11.7; 95% confidence interval [CI]: 1.57–86.7; *p* = 0.016), highlighting the importance of emerging musculoskeletal symptoms in PsA prevention [28].

Despite evidence of increased subclinical inflammation in PsO patients compared to HCs, its implications for disease progression and management are still unclear. More robust prospective studies are needed to determine whether ultrasound-detected abnormalities can reliably predict PsA progression. The recent EULAR points to consider highlight the critical role of imaging, particularly ultrasound, in detecting early musculoskeletal changes, such as synovio-entheseal abnormalities, which are hallmark indicators of PsA [17]. However, the task force cautions against using imaging alone to guide treatment in asymptomatic individuals, as such abnormalities are also common in healthy subjects. The 2023 guidance further recommends regular imaging surveillance in PsO patients with additional risk factors, such as nail involvement, obesity, or extensive skin disease, to support proactive risk management [17].

An emerging area of research investigates the role of ultrasound in identifying PsO patients who should be referred to rheumatology (e.g., primary care, dermatology clinic), often the first point of care for patients transitioning from PsO to PsA. One study compared three triage methods for PsO patients with arthralgia: physiotherapist evaluation, targeted musculoskeletal ultrasound of symptomatic joints and entheses, and PsA screening questionnaires. Combining targeted ultrasound with one of the other modalities yielded the best discriminative performance (AUC > 0.75) [29]. Similar positive results were observed when ultrasound was used in a dermatology setting [30, 31]. Overall, these findings suggest that integrating targeted ultrasound into triage strategies can enhance referral accuracy, optimizing early detection while minimizing unnecessary rheumatology consultations.

### Ultrasound in the Diagnosis of PsA

In clinical practice, ultrasound is valuable in the early diagnostic assessment of PsA, particularly in patients with suspected or subclinical disease, especially since delayed diagnosis is linked to radiographic progression and poorer outcomes [5, 32]. At PsA onset, the most common presentation pattern is peripheral arthritis, predominantly oligo-arthritis (mean number of clinically swollen joints ranging from 1.5 to 3.2) [3, 4, 18].

Various studies on early PsA (disease duration < 1–2 years) have demonstrated the higher sensitivity of ultrasound compared to clinical examination in detecting synovitis and enthesitis: subclinical synovitis was reported in up to 76% of patients with early PsA, while entheseal abnormalities with PD signal were found in 5%–14% of cases [33–35]. These findings highlight the crucial role of ultrasound in detecting subclinical inflammation and in the comprehensive assessment of early PsA.

**Fig. 2:**
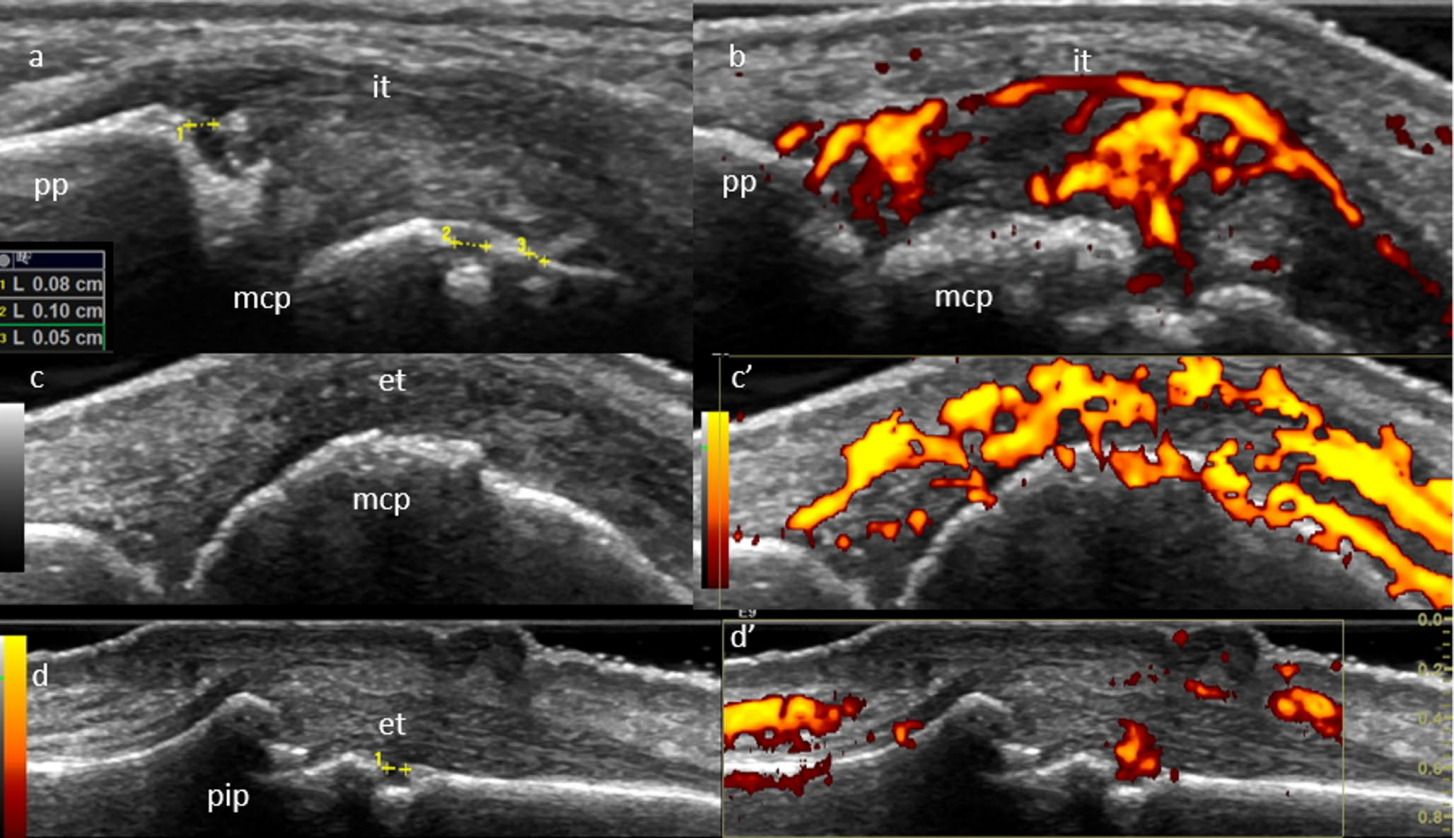
Ultrasound detection of early erosive changes in psoriatic arthritis. Legend.(**a**) Image showing multiple millimetric bone erosions (callipers) at the lateral aspect of the second metacarpophalangeal joint (longitudinal lateral view). (**b**) A “hot” bone erosion with a cortical break filled with synovial pannus (longitudinal lateral view). (**c**) Grey scale and (**c’**) power Doppler images showing close contact between synovial pannus and cortical bone with evident irregularities of the cortical bone (longitudinal dorsal view). (**d**) Grey scale and (**d’**) power Doppler images of the third proximal interphalangeal joint showing a “hot” bone erosion and active synovitis (longitudinal dorsal view). Acronyms: et: extensor tendon; it: interosseous tendon; mcp: metacarpophalangeal; pip: proximal interphalangeal

Joint erosions are common even in the early stages of PsA, indeed they are detected via ultrasound in 23%–33% of patients, especially in the wrist, second metacarpophalangeal joint, and fifth metatarsophalangeal joint [36]. High-resolution ultrasound allows visualization of millimetric cortical breaks, providing sensitive detection of early structural damage and informing timely management decisions (Fig. 2). Several studies have demonstrated the higher sensitivity of ultrasound compared to X-rays in detecting erosions, although most of this evidence comes from RA patients. X-rays may remain advantageous in certain areas where the ultrasound acoustic window is limited, such as the midfoot or posterior joints [37], highlighting the complementary roles of ultrasound and X-rays in early structural damage assessment in PsA.

One study examined the benefit of incorporating ultrasound into the Classification Criteria for Psoriatic Arthritis (CASPAR). It involved 126 patients with PsO from a Danish nationwide cohort, assessing 48 joints and 12 entheses per patient using ultrasound. Results showed that integrating sonographic findings into the CASPAR criteria significantly improved PsA classification: 66% of patients were classified as PsA when combining CASPAR and ultrasound, compared to only 35% using clinical CASPAR criteria alone and 52% of cases using ultrasound alone [38]. These data highlight both the sensitivity of ultrasound and the importance of combining clinical and sonographic assessments for early diagnosis.

Differential diagnoses of PsA can be complex, particularly when distinguishing PsA from conditions such as fibromyalgia and osteoarthritis (OA). A cross-sectional study of 848 PsO patients referred for rheumatological evaluation revealed that only 14% were diagnosed with PsA, while 37% had nonspecific arthralgia, and 49% received alternative diagnoses, predominantly OA (44%) and fibromyalgia (41%) [39]. A prospective study of 156 PsA patients, including 42 with coexisting fibromyalgia, evaluated disease activity using both clinical composite scores, such as Composite Psoriatic Disease Activity Index (CPDAI), Disease Activity index for Psoriatic Arthritis (DAPSA), and Psoriatic Arthritis Disease Activity Score (PASDAS), and comprehensive ultrasound assessment of 52 joints, 40 tendons, and 14 entheses. Patients with PsA and concomitant fibromyalgia had significantly higher clinical scores, suggesting greater disease activity, but their ultrasound scores were similar to those without fibromyalgia. Ultrasound findings correlated with clinical indices in PsA patients without fibromyalgia, but not in those with it, demonstrating that ultrasound provides a more objective measure of true inflammation and helps distinguish PsA from overlapping pain syndromes that can inflate clinical assessments [40].

OA, especially its erosive form, may also mimic PsA, particularly when affecting the proximal and distal interphalangeal joints [4]. Ultrasound may detect inflammatory features such as synovitis or erosions within a single affected joint; however, this alone cannot reliably distinguish OA from PsA. The distinction becomes clearer when evaluating other joints and entheses: PsA typically shows patterns of inflammation such as enthesitis, tenosynovitis, dactylitis, and peritendinitis at sites beyond the initially affected joint, which are generally absent in OA. Importantly, although ultrasound cannot definitively differentiate PsA from OA, detecting inflammatory signs or central bone erosions in OA—particularly in its erosive form—may have implications for disease management, including the consideration of conventional disease-modifying anti-rheumatic drugs (DMARDs) in more inflammatory cases [41].

Although PsA and RA share features such as synovitis, ultrasound can help differentiate them by evaluating the pattern and distribution of inflammation. In RA, synovitis is generally a primary, intra-articular process, whereas in PsA it is considered to arise from entheseal inflammation, consistent with the “synovio-entheseal complex”. Extra-synovial manifestations, such as enthesitis, tenosynovitis, and peritendinitis, are more common in PsA and provide important distinguishing clues. These lesions frequently occur at mechanically stressed sites, including annular pulleys, extensor tendons, and adventitial bursae, which have fibrocartilaginous structures similar to entheses [42]. Flexor tendon tenosynovitis is the second most frequent ultrasound finding in PsA after synovitis, and although peritendinitis of the finger extensor tendon is less common, it is considered relatively specific for PsA [43, 44]. By assessing both intra-articular and extra-synovial involvement, ultrasound offers an objective, non-invasive method to distinguish PsA from RA, even when synovitis appears morphologically similar in both conditions [45, 46].

**Fig. 3:**
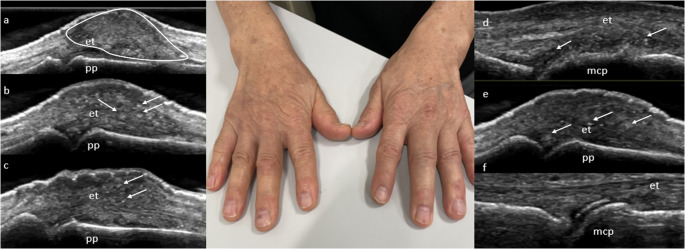
Ultrasound in the differential diagnosis of psoriatic arthritis. Legend. A man was referred to rheumatology with polyarticular joint pain and swelling. He had a previous diagnosis of gout (serum urate <400 µmol/L on allopurinol 300 mg/day) and a family history of PsO, raising suspicion for PsA. No cutaneous tophi were present. (**a–c, d**) Dorsal longitudinal images of the metacarpophalangeal region showing hyperechoic deposits within and adjacent to the extensor tendon and over the proximal phalanx, consistent with monosodium urate (MSU) crystal deposition, including cloud-like deposits (outlined in white in figure **a**) and small urate aggregates, which are pointed out by arrows in **b** and **c**. Dorsal longitudinal images of the metacarpophalangeal joints demonstrate intra-synovial MSU crystal aggregates (arrows in **d**), intra-tendinous and peri-tendinous deposits (**e**) and crystal deposition along the cartilage surface consistent with a double contour sign (**f**). Acronyms: et: extensor tendon; mcp: metacarpophalangeal; pp: proximal phalanx

Crystal-induced arthritis should also be considered in the differential diagnosis of PsA, particularly because both conditions often present with mono/oligoarthritis. Ultrasound has demonstrated excellent ability in diagnosing crystal-induced monoarthritis, thus aiding in the differential diagnosis from other types of arthritis. In a recent study in patients with acute mono/oligoarthritis, ultrasound findings showed specificity > 90% and good sensitivity for both gout and crystal pyrophosphate deposition disease (CPPD) using a targeted scanning protocol of two joints bilaterally (gout: knee and first metatarsophalangeal joint; CPPD: knee and wrist) in addition to the symptomatic joint, achieving overall diagnostic accuracy greater than 90% for both conditions [47]. The differential diagnosis between PsA and gout remains challenging, especially as many studies have reported cases in which the two conditions coexist (referred to as “Psout”) [48]. Hyperuricemia is indeed more common in patients with PsO or PsA compared to the general population. The presence of hyperuricemia in PsA patients could potentially influence patient characteristics and treatment outcomes, but stronger data is needed to confirm this relationship [49, 50]. Similarly, in patients with polyarticular involvement, where clinical distinction between PsA and crystal-induced arthritis may be particularly difficult, ultrasound is extremely helpful, as it can visualize urate (or CPPD deposits) and distinguish between the two conditions, with very important implications for patient management (Fig. 3).

Finally, it is important to acknowledge that entheseal ultrasound abnormalities may be influenced by non-inflammatory factors. Obesity, increasing age, and metabolic comorbidities such as diabetes mellitus and hypercholesterolaemia are associated with a higher prevalence of entheseal structural changes, including enthesophytes, which may confound interpretation in PsA and PsO [51–53]. In addition, recent mechanical loading and physical exercise can transiently increase PD signal at entheses [54]. These factors highlight the need to interpret entheseal ultrasound findings within the broader clinical and metabolic context, particularly when assessing subclinical disease or differential diagnoses.

### New Insights into the Ultrasound Assessment of Enthesitis in PsA

Enthesitis—inflammation at sites where tendons, ligaments, or joint capsules insert into bone—is a hallmark feature of spondyloarthritides (SpA), including PsA [55].

In PsA, enthesitis plays a central role in disease pathophysiology and is closely linked to clinical symptoms and long-term outcomes, with prevalence estimates ranging from 35% to 74%, depending on diagnostic criteria and study populations [56–58]. Clinically, enthesitis typically manifests as persistent pain, stiffness, and functional impairment, significantly affecting quality of life and frequently guiding therapeutic decisions, including escalation to biologic therapies when conventional treatments are insufficient [59, 60].

Traditionally, assessment relies on physical examination using standardized scoring systems such as the Leeds Enthesitis Index (LEI) and the Maastricht Ankylosing Spondylitis Enthesitis Score (MASES) [61, 62]. However, these methods are limited by their reliance on subjective pain, which can be influenced by mechanical factors or conditions such as fibromyalgia, making it challenging to distinguish true inflammatory enthesitis from non-inflammatory causes of pain. Over the past two decades, ultrasound has emerged as a sensitive, non-invasive, and dynamic tool for entheseal evaluation, providing detailed visualization of both anatomy and pathology [11, 63]. Several ultrasound scoring systems, including GUESS, the Madrid Sonographic Enthesis Index (MASEI), and GRAPPA-based methods, have been developed to standardize imaging assessment across different entheses and lesion types [64].

The OMERACT ultrasound task force further refined definitions of key lesions, distinguishing features of active inflammation, such as entheseal thickening, hypoechoic areas, and PD signal, from structural damage, including enthesophytes, calcifications, and bone erosions [65]. Enthesitis is defined as a hypoechoic and/or thickened tendon insertion within 2 mm of the cortical bone, with PD signal if inflamed, whereas structural lesions reflect chronic or prior disease. Thickening and hypoechoic changes are not entirely specific to inflammatory disease and may be observed in fibromyalgia, OA, obesity, or physically active individuals [12]. Moreover, reliability of certain features can vary, as shown in web-based scoring exercises, highlighting the importance of standardized training and reference image atlases [66].

Ultrasound has been shown to help differentiate inflammatory enthesitis from other causes of tenderness. In the ULISSE study, clinical examination identified enthesitis in 92% of patients with fibromyalgia; however, ultrasound findings were more specific for PsA-related enthesitis, with only 75% showing both tenderness and ultrasound-detected entheseal involvement [67].

**Fig. 4:**
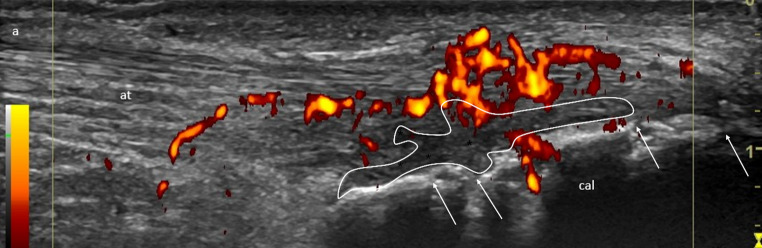
Ultrasound detected enthesitis in psoriatic arthritis. Legend. (**a**) Image showing active, erosive enthesitis at the calcaneal insertion of the Achilles tendon. Note the presence of entheseal thickening, hypoechoic areas (outlined by white line), and power Doppler signal at the enthesis, including filling of cortical bone erosions pointed out by arrows (dorsal longitudinal view). Acronyms: at: Achilles tendon; cal: calcaneal bone

The Defining Enthesitis on Ultrasound in Spondyloarthritis (DEUS) initiative evaluated the reliability and clinical relevance of the OMERACT lesions of enthesitis, and developed the DEUS Enthesitis Index (DEI), a combined clinical-ultrasound scoring system [68]. DEUS studies demonstrated that features such as PD signal and bone erosions strongly distinguish SpA-related enthesitis from mechanical or fibromyalgia-related tenderness, with the Achilles tendon identified as the most discriminative enthesis in over 400 SpA patients and nearly 300 disease controls [69]. An example of active, erosive enthesitis of the Achilles tendon in a patient with PsA is shown in Fig. 4.

DEUS also highlighted marked discordance between clinical and ultrasound findings, with approximately 70% of clinically tender entheses showing no ultrasound evidence of active inflammation, and around 15% of non-tender sites demonstrating subclinical disease [70]. This discordance may reflect differences in tissue biology, whereby pain at the enthesis can arise from mechanical load or microdamage in a structure with limited vascularity, meaning that symptoms and inflammatory activity may not fully overlap, in contrast to the synovium in RA. In addition, these observations underscore that clinical signs may be influenced by mechanical factors or central sensitization, whereas ultrasound can detect subclinical disease that may be clinically relevant and guide treatment strategies.

Recognizing the complementary strengths of clinical and imaging assessments, the DEI was the first tool designed to combine both modalities for the evaluation of enthesitis [71]. The DEI assesses five key lower-limb entheses bilaterally, scoring each site for clinical tenderness and ultrasound-detected lesions to generate a total score ranging from 0 to 20. This composite approach allows the DEI to capture both patient-reported symptoms and objective imaging findings, correlating with markers of disease activity, structural damage, and systemic inflammation. By integrating these dimensions, the DEI provides a more comprehensive and reliable measure of enthesitis than either clinical or imaging assessment alone. Its sensitivity to change, however, still requires further demonstration in longitudinal studies.

Beyond active inflammation, ultrasound assessment of structural damage provides insight into SpA phenotypes. DEUS studies showed a high prevalence of enthesophytes, calcifications, and erosions, with distinct patterns in PsA versus axial SpA [72]. Enthesophytes were most common in PsA and associated with clinical enthesitis and PsO, erosions reflected inflammation-driven disease, and calcifications correlated with age and metabolic comorbidities. These findings highlight the potential of ultrasound-detected lesions to inform disease severity and phenotypic stratification in SpA.

Ongoing studies, such as the DUET project, are further exploring these findings, aiming to improve ultrasound standardization and establish its potential use in clinical practice, ensuring more precise and consistent assessment of enthesitis in SpA patients [73]. In addition, increasing attention has been directed toward assessing smaller entheses, particularly those in the hands, which will be discussed in a dedicated paragraph.

### Patients with established PsA

#### Assessing Response to Therapy

Ultrasound is a sensitive tool for evaluating therapeutic response in patients with established PsA. Studies consistently demonstrate that ultrasound can detect changes very early after treatment initiation, sometimes within the first week, and that these changes correlate with improvements in patients treated with DMARDs [74–78]. In one study including 25 patients (9 with PsA and 16 with SpA), ultrasound detected significant improvements in both the MASEI and OMERACT PD scores at three- and six-month follow-up [75]. A separate study of 30 patients corroborated these findings and further showed that synovial PD improvements were more pronounced than entheseal PD responses at both three and six months [76]. Additionally, in a comparative study of TNF inhibitors and secukinumab involving 80 PsA patients, ultrasound demonstrated differences in therapeutic response that were not apparent on clinical assessment [77]. Specifically, the change in the active components of the MASEI scores and PD activity at the enthesis were significantly higher in patients receiving TNF inhibitors, highlighting the ability of ultrasound to detect subtle treatment effects.

#### Ultrasound Remission

Ultrasound has proven its potential as a sensitive tool for assessing response to therapy, as described in the previous paragraph, but also for detecting residual disease activity: indeed, one study showed that among PsA patients in clinical remission after six months, ultrasound still revealed persistent subclinical synovitis and enthesitis [79]. It is important to note that, similar to clinical remission, there remains no consensus on the definition of “ultrasound remission.” Proposed definitions range from a PD score of 0 to a PD score ≤ 1 for any lesion, resulting in reported ultrasound remission rates between 17% and 69% depending on the definition used, and often showing discordance with clinical remission rates [80–84]. From this perspective, establishing shared thresholds for ultrasound remission that account for the multiple domains of the disease represents a key priority for future research.

Therefore, for several reasons, clinical and ultrasound remission may not coincide. A recent systematic review specifically examined the role of ultrasound in the management of PsA [11], proposing two treatment algorithms based on the type of response achieved. In patients showing a good clinical response, concordance between clinical and ultrasound findings supports a gradual tapering of therapy, whereas persistent ultrasound activity suggests maintaining treatment to reduce the risk of relapse. Conversely, in patients with poor clinical response, treatment intensification is recommended if ultrasound confirms active inflammation; when there is discordance between clinical and ultrasound findings, it is instead appropriate to consider alternative causes of non-response.

**Fig. 5:**
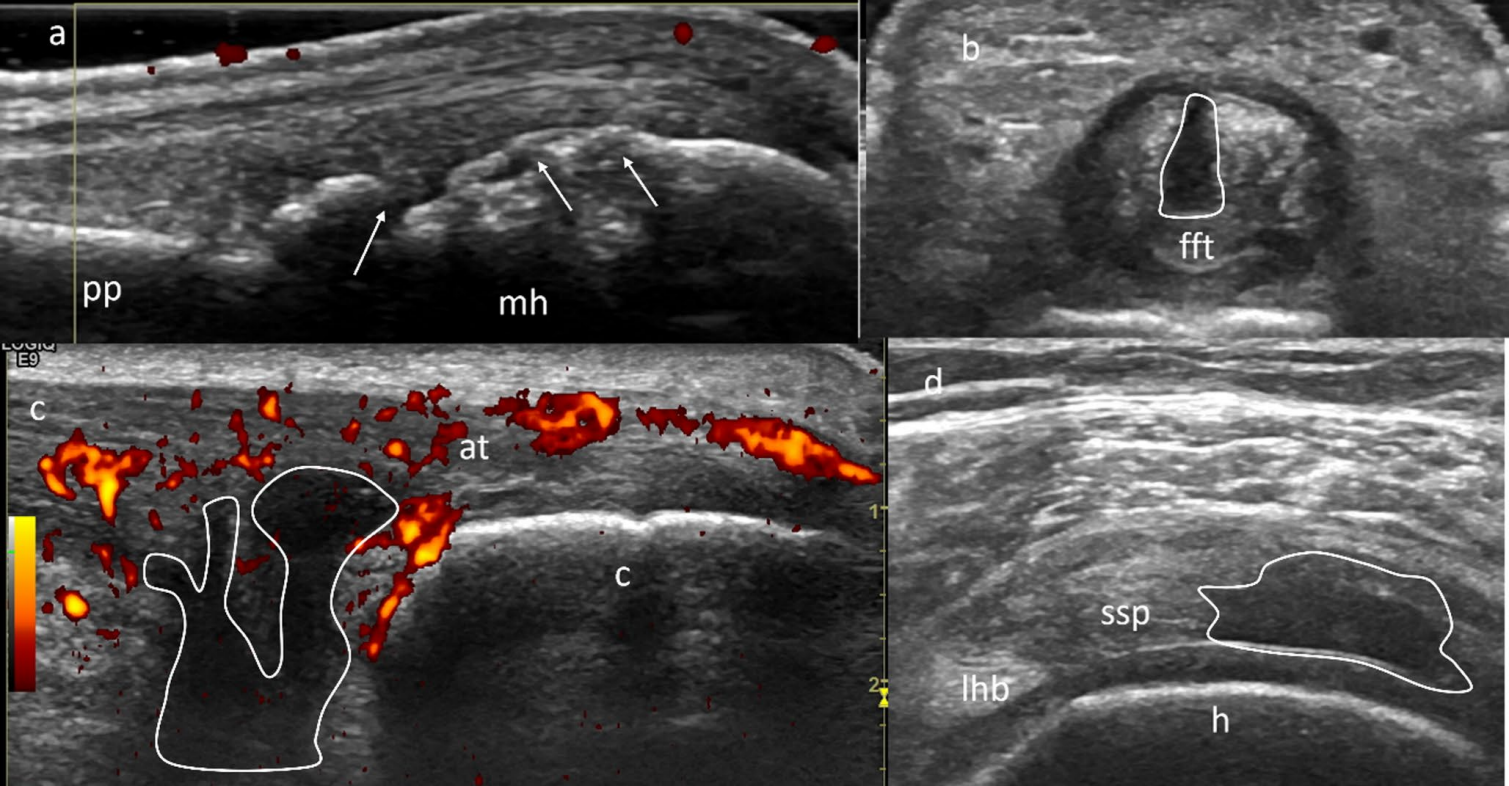
Structural damage at joint and tendon level in patients with PsA. Legend. (**a**) Dorsal longitudinal image showing severe structural damage at the metacarpal head with multiple bone erosions (arrows) and malalignment in a patient with ongoing pain, without evidence of active synovitis. (**b**) Transverse ultrasound image of the second finger flexor tendons demonstrates focal hypoechoic changes (outlined by white line) within the superficial finger flexor tendon consistent with a partial-thickness tear. (**c**) Longitudinal ultrasound image of the Achilles tendon demonstrates preinsertional focal fiber disruption of the deep tendon layers (outlined by white line), consistent with a partial-thickness tear (subsequently confirmed on MRI). Note the presence of surrounding power Doppler signal within the Achilles tendon fibers. (**d**) Transverse image of the supraspinatus tendon showing a complete loss of normal fibrillar pattern across the tendon thickness (outlined by white line), consistent with a full-thickness tear. Acronyms: at: Achilles tendon; c: calcaneal; fft: finger flexor tendon; mh: metacarpal head; ssp: supraspinatus tendon; lhb: long head of biceps tendon; h: humerus; pp: proximal phalanx

These may include factors associated with patient-evaluator global assessment discordance, such as pain, fatigue, depressive symptoms, and impaired quality of life, independent of objective ultrasound inflammation [85]. For example, as illustrated in Fig. 5, ultrasound can identify structural lesions, such as joint structural damage (i.e., malignment, bone erosions, osteophytosis) or tendon rupture, which may explain persistent pain independently of inflammatory activity and require a different management strategy.

#### Prognostic Factors Identified by Ultrasound

Several studies indicate that ultrasound is not only an important tool for the diagnosis and monitoring of PsA, but also a valuable instrument in identifying prognostic markers and risk factors for flares and worse disease outcome including radiographic progression. In a cohort of 54 PsA patients in clinical remission, PD-detected synovitis proved to be a strong predictor of short-term flares [86]. Similarly, a study of 36 patients showed that joints with baseline ultrasound-detected synovitis were more resistant to therapy and that even non-tender, non-swollen joints with subclinical synovitis had a higher risk of developing symptoms over time [87]. A prospective cohort study of 107 patients further demonstrated that a higher ultrasound erosion score was associated with earlier drug discontinuation [88].

Enthesitis have been identified as a predictive factor for erosions [57, 89], together with dactylitis [90]. A further study conducted on 150 patients, followed for 18 months, found that tenosynovitis, more than synovitis, was an independent predictor of both disease persistence (OR 6.6; *p* = 0.002) and erosions [91].

#### Ultrasound as a Guiding Tool for Difficult-to-Manage and Treatment-Refractory PsA

The management of PsA that remains symptomatic despite multiple lines of therapy is challenging, and distinguishing true treatment refractoriness from non-inflammatory disease is critical. In this context, EULAR defines difficult-to-manage (D2M) PsA as the persistence of symptoms despite several therapies, while treatment-refractory (TR) PsA refers to the subset of patients with objectively confirmed ongoing inflammation [92]. Ultrasound is central to this distinction, enabling clinicians to differentiate active inflammatory disease from symptoms driven by non-inflammatory mechanisms such as pain sensitization, comorbidities, or irreversible structural damage.

This inflammatory versus non-inflammatory paradigm was first developed in RA, where difficult-to-treat disease has been stratified according to the presence or absence of ultrasound-detected synovitis, revealing that up to 40% of refractory cases lack objective inflammation and are frequently associated with comorbidities such as obesity, osteoarthritis, and fibromyalgia [93]. Similar observations have recently been reported in PsA, where an analogous distinction has been proposed between patients with persistent inflammatory disease and those with predominantly non-inflammatory symptoms [94]. Unlike RA, PsA exhibits greater heterogeneity, making the distinction more complex. Nevertheless, ultrasound remains a key tool for differentiating these states and potentially avoiding unnecessary treatment escalation. In a cohort of difficult-to-treat PsA patients with active disease by composite measures (DAPSA > 14), objective inflammatory findings were identified in approximately 60% of cases, while the remaining patients showed little or no ultrasound evidence of inflammation [95]. Patients with inflammatory disease were more likely to exhibit swollen joints, dactylitis, and nail PsO, whereas those without inflammatory findings predominantly presented with tender points, painful entheses without PD signal, and marked discordance between patient-reported and physician-assessed disease activity.

In this setting, ultrasound provides critical insight not only by confirming or excluding active synovitis or enthesitis, but also by localizing the involved anatomical structures and identifying alternative contributors to symptoms, such as osteoarthritis or microcrystalline disease. Imaging may also complement other modalities, including MRI, which can detect peri-entheseal osteitis not visible on ultrasound [96]. Although the therapeutic implications of this stratification remain to be fully defined, these findings underscore the potential of imaging to avoid inappropriate treatment escalation and to support a more precise and individualized management approach in difficult-to-treat PsA.

### Ultrasound Scoring Methods for PsA

Due to its high sensitivity in detecting inflammatory musculoskeletal lesions, ultrasound has become also an essential tool for both diagnostic and monitoring scoring in PsA. Developing a composite score capable of assessing all aspects of PsA is particularly challenging, given the heterogeneity of lesions in the disease. Several scoring systems have indeed been proposed, each focusing on different disease domains. In this section, we will review the most recent scores, categorized by the domains they address.

For the assessment of synovitis, the most commonly used score is GLOESS, a composite severity score combining B-mode and Doppler mode. Initially developed for RA, it is now also applied to PsA, as synovitis is a common feature in both conditions, and ultrasound is highly sensitive and specific for its detection [74, 97].

In the assessment of enthesitis, several newer scores have been proposed. In 2019, GRAPPA developed the “DUET” (GRAPPA Diagnostic Ultrasound Enthesitis Tool), a composite score that analyzes 16 entheseal sites to standardize the ultrasound diagnosis of enthesitis [73]. Like other studies, it emphasized that entheseal thickening, hypoechogenicity, and PD signal should be considered indicative of “active enthesitis” whereas calcifications, enthesophytes, and bone erosions are markers of “structural damage.” This score shows high inter-reader reliability [98]. Another enthesitis score is the Belgrade Ultrasound Enthesitis Score (BUSES). It is a cumulative score that assigns points based on the presence of enthesis thicknening, hypoechogenicity, enthesophytes, PD signal, and erosions across 10 enthesal sites. The total BUSES score is 132, and a value ≥ 7 is highly specific for the diagnosis of SpA [99]. It has demonstrated excellent reliability. Another enthesitis score is the Cortical-Entheseal Remodeling Tuscany Ultrasonographic Score (CERTUS), which quantifies cortical-entheseal remodelling through grey-scale imaging and integrates PD (CERTUS-PD) for detecting vascular signals. This score evaluates enthesophytes and erosions across 12 peripheral entheses, offering high specificity for PsA-related enthesitis, even in subclinical PsA-related enthesitis [100]. It has demonstrated excellent correlation with validated scores like BUSES and clinical markers of disease activity [100]. As mentioned earlier, the DEI is the first score to combine ultrasound and clinical evaluation for the assessment of enthesitis in SpA [71].

Additionally, a composite ultrasound score, named PsASon, has been developed to assess both inflammatory and structural lesions in PsA. This comprises two scoring methods: the PsASon13 (unilateral joints) and PsA-Son22 score (bilateral joints), assessing synovitis, tenosynovitis, enthesitis, and erosions. Both scores have demonstrated sufficient construct validity, reliability, and sensitivity to change [101]. A very recent work has introduced the Ultrasound Psoriatic Arthritis (UPsA) scores, designed to quantify both inflammatory activity and structural damage across multiple musculoskeletal domains. The UPsA Activity Score demonstrates construct validity and moderate-to-high sensitivity to change, while the UPsA Damage Score correlates with radiographic indices of structural progression. These composite scores are particularly suited for clinical trials, providing standardized, reproducible, domain-specific imaging outcomes and complementing clinical assessment [102].

**Fig. 6:**
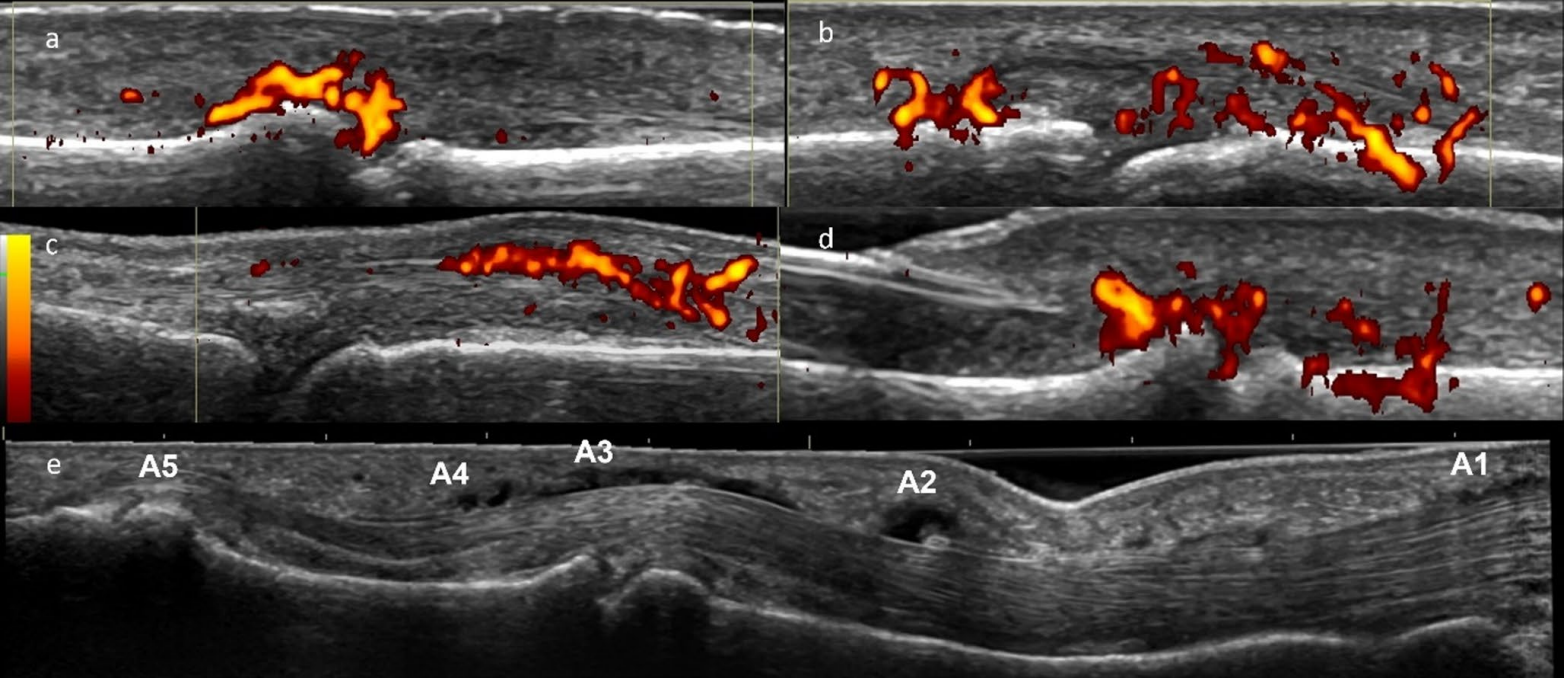
Small hand enthesitis in patients with PsA. Legend. (**a**) Active enthesitis of the finger extensor tendon central slip at its insertion into the middle phalanx acquired using a dorsal longitudinal scan at the proximal interphalangeal joint level; (**b**) Radial collateral ligament enthesitis of the index finger, visualized at its insertion on the proximal phalanx using a lateral longitudinal ultrasound scan. (**c**) Peritendinitis of the finger extensor tendon, acquired using a longitudinal dorsal scan at the metacarpophalangeal joint level; (**d**) Distal interphalangeal joint enthesitis of the extensor tendon insertion into the distal phalanx, imaged with a dorsal longitudinal scan at the distal interphalangeal joint level; **(e)** Dactylitis of the middle finger with inflammation of multiple flexor pulleys, and tendon sheath effusion, acquired using a volar longitudinal scan along the entire digit.

In addition to the OMERACT ultrasound definitions for synovitis and enthesitis [103], several ultrasound definitions and scoring approaches for dactylitis—another hallmark feature of PsA have been proposed and investigated [104, 104]. Zabotti and colleagues proposed an ultrasound scoring system for dactylitis, named the DACTylitis glObal Sonographic score in PsA (DACTOS). In this score, elementary lesions of dactylitis were evaluated through a Delphi exercise involving 12 experts, reaching consensus on scoring criteria. It has demonstrated moderate to excellent reliability for ultrasound-assessed lesions [105]. A new scoring system for dactylitis, the GLobal OMERACT Ultrasound DActylitis Score (GLOUDAS), was recently developed to evaluate synovitis, tenosynovitis, and enthesitis [106]. Unlike DACTOS, which focuses primarily on scoring the affected digit as a whole, GLOUDAS includes 12 mini-entheses in the fingers (Fig. 6) which had not been used in previous scoring systems, which are identified through histological studies and a Delphi process. The scoring system was further validated through an additional Delphi process to establish all other ultrasound components of dactylitis. GLOUDAS demonstrated excellent intra-observer and good inter-observer reliability, providing a reliable tool for both clinical and research applications in psoriatic dactylitis [106].

## Conclusion

**Fig. 7:**
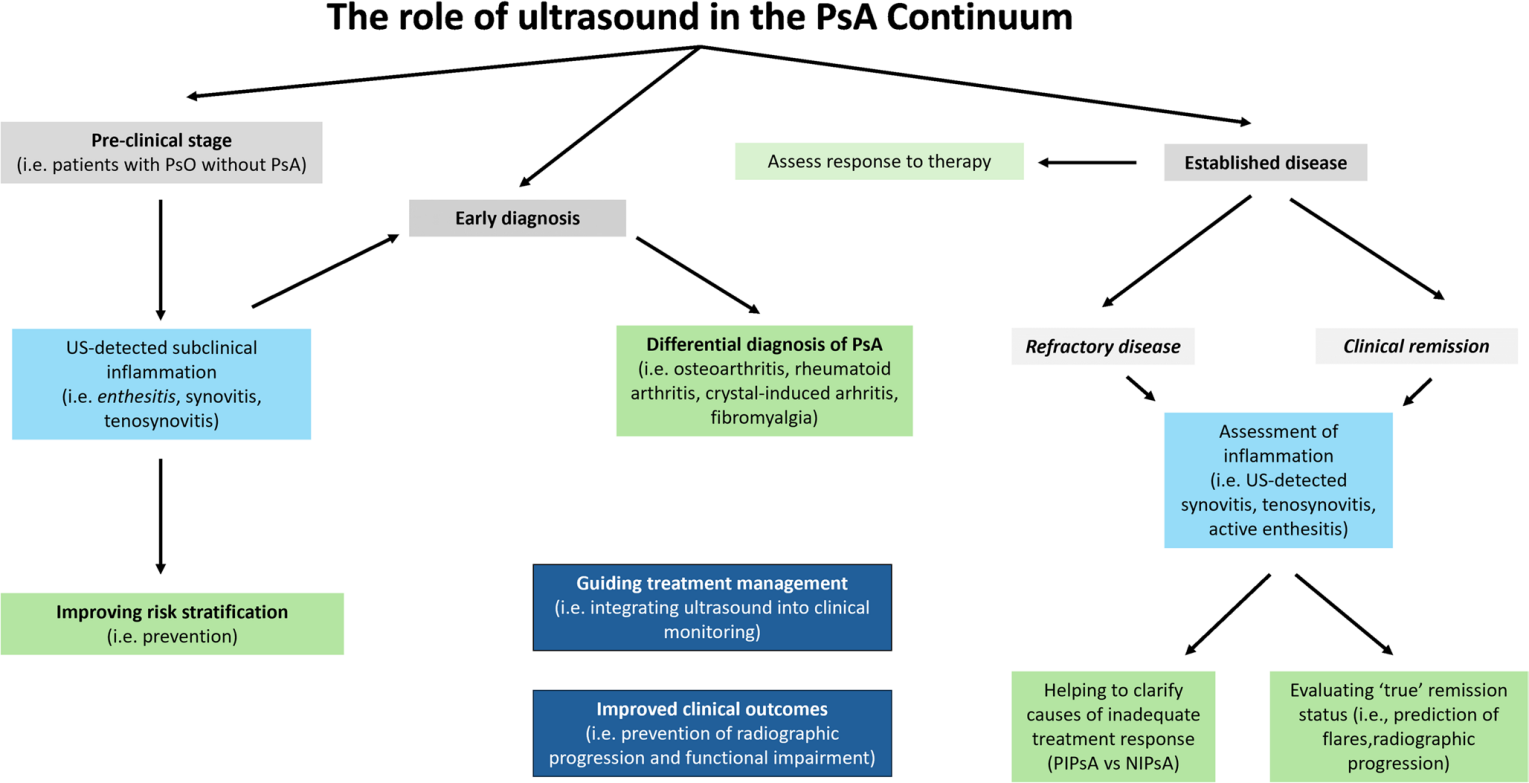
The potential uses of ultrasound in the psoriatic arthritis continuum. Legend. The figure illustrates the potential value of ultrasound across the PsA continuum, from preclinical stages to established disease. In individuals with PsO without PsA (preclinical stage), ultrasound detection of subclinical inflammation, including enthesitis, synovitis, and tenosynovitis, can improve risk stratification and identify patients at higher risk of developing PsA. In early or undifferentiated disease, ultrasound may assist in early diagnosis and guide management during the initial phases of disease, when timely intervention may prevent structural progression and functional impairment. In established PsA, helps assess response to therapy. In addition, ultrasound may help clarify causes of inadequate treatment response, and differentiate persistent inflammatory PsA (PIPsA) from non-inflammatory PsA (NIPsA); on the other side, it applies to disease remission, where ultrasound can detect subclinical inflammation (i.e., assess true remission status), and predict flares. Across all stages, ultrasound supports integration into clinical management and contributes to improved long-term outcomes

This review highlights the main applications, strengths, and clinical practice implications of ultrasound use across the PsA continuum (Fig. 7). We also discuss the current limitations of this technique and outline future directions for research and clinical implementation. Ultrasound is valuable even in preclinical disease, providing insight into the evolution from PsO to PsA by detecting subclinical synovitis, enthesitis, and tenosynovitis, as well as early structural changes such as entheseal erosions. Although the prognostic and clinical implications of these subclinical findings are not yet fully defined, particularly their ability to predict progression to clinically overt PsA, they may support disease interception strategies and help identify patients who warrant rheumatologic assessment.

Ultrasound also plays an important role in differential diagnosis, improving discrimination between PsA and other conditions, including fibromyalgia and other inflammatory arthritides. In disease monitoring, it can resolve discrepancies between clinical symptoms and inflammatory activity, thereby informing decisions on treatment escalation, de-escalation, or maintenance.

Emerging applications include novel scoring systems, assessment of previously underexplored structures such as small hand entheses, and improved characterization of features such as dactylitis. Ongoing research and greater standardization of ultrasound protocols are essential to define clinically meaningful targets and fully realize the potential of ultrasound in personalized PsA management.

## Data Availability

No datasets were generated or analysed during the current study.
